# Supplementary material for: Detecting epinephrine auto-injector shortages in Finland 2016–2022: Log-data analysis of online information seeking
Source: PLoS One. 2024 Apr 11;19(4):e0299092. doi: 10.1371/journal.pone.0299092 (PMC11008843; doi:10.1371/journal.pone.0299092)
Supplement: Attachment — Submitted filename: Response To Reviewers.docx [file pone.0299092.s002.docx]

Dear Prof. Radoslaw Wolniak and Reviewers #1 and #2,

Thank you for your excellent suggestions in order to improve our manuscript. We have now provided the revisions described below.

Kind regards,

Dr. Milla Mukka

Response to Reviewers

Reviewer #1: Dear Authors,
Congratulations for your interesting research. I have some suggestions on how to make your text more attractive

**Response:** Thank you for your comment.

In the introduction, you write about the previous research. I suggest that you briefly explain what the research was, when it was conducted, perhaps refer to publications if published.

**Response:** Thank you for your excellent comment. We have now added more information on our previous studies by providing details, years, and publications [20-24].

In the introduction, please state the originality of the material presented. I suggest in the introduction specify the methodology of the research. The diagnosis itself should indicate the novelty of the results and the publication of the considerations in scientific journals. The current state of the research field should be thoroughly reviewed and key publications should be cited.

**Response:** We have now revised Introduction by adding subsections and also the methodology of the research. We have searched and reviewed the key publications of the research field.

Some amendments in the format are due to admit publication. The main problem is the epistemological structure (why the article was conceived and how the study was developed). I suggest the following structure of objectives: (i) research gap; (ii) purpose of the article; (v) assumptions or hypo; and (vi) research method. This structure must appear in the introduction.
The research gap must be created by a systematic literature review that provides 'holes' in the state of knowledge on the topic. I believe that a full review should not be done, but an analysis of about 5-8 studies on the topic under discussion. You can find some examples, which will show the relevance of the issue, as it is indeed a topic of current, relevant research. At the end of the justification you should write something like: According to what we were able to find, there are no studies referring and reporting on ... With this you have therefore proven that the issue is relevant, and you have also proven that your study does indeed fill a research gap.

**Response:** We thank the reviewer for this suggestion. We have now revised Introduction by using the structure (subsections), namely “Medicine shortages”, “Auto-injectors”, “Online information seeking”, as well as the suggested subsections “Research gap”, “Purpose of the study, and “Hypotheses”. We used the subsection of “Literature search” instead of “Research method” where we describe the searching method of literature (methodology). We performed the literature search from the four databases in order to find the published articles on epinephrine auto-injector shortages. The key publications found were reviewed and analyzed. At the end of Introduction, we justify our study as suggested.

In the discussion, I suggest that describe the significance of the research and its impact on the wider field, show how the information obtained can be further used

Conclusions must be clearly and unambiguously linked to the results of the study. Their theoretical and practical implications should be indicated

**Response:** Thank you for your comment. We have added information on the meaning of our research by describing how our study results can be used in detection of epinephrine shortages. We have now revised the conclusion section and added information in the discussion. We have also described the achievements of the general and two aims of our study, as well as shown the reason why the analysis was done (Reviewer #2 comment). In Conclusion, we have separated this section in two, in order to show the theoretical and practical implications on the wider field as well as showing the future aspects.

The methodology should indicate the time period that was considered in collecting data on drug shortages.

**Response:** We performed the literature search in order to review the research field of epinephrine auto-injector shortages, yielding four key publications during 2016-2020, and one in 2005. Thus, the data were collected in our study during 2016-2022. We have mentioned this in Introduction.

Good luck!

Reviewer #2: PLOS One review 10 July 2023
Dear Authors,
The paper is interesting. Drug shortage is an important issue. The analysis is made of improvement. As a reviewer, I have a few comments:

**Response:** Thank you for your comment.

Note 1: Keywords: In my opinion there are too many keywords.

**Response 1**: Thank you for your opinion. We have limited the number of keywords.

Note 2: Introduction: several references for one sentence in not good: an example (60-61): Medicine shortages may cause harm and even life-threatening situations for patients encountering medication errors and adverse events [1,2,4-6]. In my opinion, authors have to write more about the events… along for each reference.
Reference in sentence (62) In different countries, the reasons for 63 medicine shortages may vary [4,7]…
Many single sentences have their own references, this is not good. Authors need to write more about what each reference contributes to their research topic.

**Response 2**: Thank you for your comment. We have now written more text with less references embedded in one sentence as suggested. Literature is introduced more with longer sentences forming two separate paragraphs (“Medicine shortages”).

Aim (97 etc) there are two aims in one. I propose to prepare separate aim in the form; General aim:
And RQ: research questions: RQ1:… RQ2:…..

**Response**: Thank you for your excellent proposal. We have now clarified the aim and research questions in text. This revision is placed under the title of “Purpose of the study” in Introduction. The separation of study aims is also revised in Abstract.

Note 3: Setting. In my opinion is OK but I think there should be more information about the situation in the country regarding the drug distribution system, maybe what scheme....

**Response 3**: We have clarified the medicine distribution system in Finland in the setting.

Note 4: analysis without comments

**Response 4:** We have added more discussion/comments on our results/analysis in the discussion section. Also, we have added the subsection of “Interpretation of results” in order to discuss and comment our study results.

Note 5. Conclusion, too short. Describe the aims achieved, show why analysis was done.

**Response 5:** Thank you for your comment. We have now revised the conclusion section. We have also described the achievements of the general and two aims of our study, as well as shown the reason why the analysis was done.
