# Supplementary material for: Detecting epinephrine auto-injector shortages in Finland 2016–2022: Log-data analysis of online information seeking
Source: PLoS One. 2024 Apr 11;19(4):e0299092. doi: 10.1371/journal.pone.0299092 (PMC11008843; doi:10.1371/journal.pone.0299092)
Supplement: Attachment — Submitted filename: ResponseToReviewers.docx [file pone.0299092.s003.docx]

Dear Prof. Radoslaw Wolniak and Reviewer #2,

Thank you for your suggestions in order to improve our manuscript. We have now revised our paper by providing the answers below. Grammar has also been revised throughout.

Kind regards,

Dr. Milla Mukka

Response to the Editor and Reviewer #2

Journal Requirements: Please review your reference list to ensure that it is complete and correct.

**Response:** Thank you for your reminder to reference list. We have checked and updated the reference list on pages 22-26.

Reviewer #2: 
The subject is narrow, but we flow. Your analysis is good, but I have comments:

**Response:** Thank you for your comment.

Note 1. Why there is no Literature review section. Such section should be before Materials

and methods.

**Response 1:** Thank you for your comment. We have done the literature search and added it before Materials and Methods (on pages 6-7). The section title “Literature search” is now changed into “Literature review”.

Note 2. Section: Setting is too short.

**Response 2:** Thank you for your notice. We have made this section longer by adding more information on Duodecim Publishing Company Ltd, the number of healthcare professionals in Finland, and Kela. Now, the setting section comprises three paragraphs on pages 8-9.

Note 3: Record numbers: now is 34829, should be: 34,829

**Response 3:** Very good notice. We have made changes in the record numbers in the tables.

Note 4: Fig 1-3 Blurred, fuzzy fonts

**Response 4:** Thank you for your comment. We have now checked all the figures and uploaded them in the PACE system. PACE accepted the figure files in resolution based on the PLosOne requirements.

Note 5. Conclusion too short. Please write about reserach limitation and describle problem persented in your research.

**Response 5:** Thank you for your notice. We have discussed the research limitations in its own section “Strengths and limitations” on pages 17-18. More information on the limitations and possible problems of our study has been added. New information has also been added in Conclusion on pages 19-20. Now, the conclusion section comprises five paragraphs.
