# Supplementary material for: Detecting epinephrine auto-injector shortages in Finland 2016–2022: Log-data analysis of online information seeking
Source: PLoS One. 2024 Apr 11;19(4):e0299092. doi: 10.1371/journal.pone.0299092 (PMC11008843; doi:10.1371/journal.pone.0299092)
Supplement: Attachment — Submitted filename: Response to Reviewers.docx [file pone.0299092.s004.docx]

Dear Academic Editor Muhammed Shahzad Aslam, Basant Adhikari, and Reviewer #4,

Thank you for your suggestions in order to improve our manuscript. We have now revised our paper by providing the answers below. We will be happy to do further revisions in the manuscript to move forward to publication in PlosOne.

Kind regards,

Dr. Milla Mukka

Response to the Editor

Additional Editor Comments:

In the method section, it is advisable to provide clear definitions for terms such as "Auto-injectables" and "Shortage of auto-injectables." Additionally, specify how the shortage of auto-injectables is defined according to Fimea.

**Response:** Thank you for your comments. We have checked the correct terms in the literature, and we think that they are “auto-injectors” and “shortage of auto-injectors”. We have provided the definitions for them in the Materials and Methods section. Also, the definition from Finnish Medicines Agency has been provided in this section.

In the Discussion section, the following points are expected from the authors:

a. Elaborate further on possible reasons for the findings related to the first and second research questions.

b. Explore the association between 'Searching' and 'Shortage': specify the nature of this association, whether it might be temporal or a spurious correlation.

**Response:** We thank for these comments.

a. We have elaborated further on possible reasons for findings of research question one and two. First, more information on professionals’ searches has been provided in Discussion. Second, more information on the relation of citizens’ article openings and shortages, as well as summertime and lesser prescriptions, has been added in Discussion.

b. We have now specified the association as “temporal relation” in Discussion and elsewhere in our paper. However, our data on searches and shortages are not able to provide statistical analysis, such as correlation, so we use “visual relation” or “temporal relation” between searches and shortages explored in the figures. We found temporal relation between searches and shortages, thus we mention “visually similar seasonal patterns” in Discussion. Also, this has been added in the Limitations section.

Reviewer #4
The contents of the research article are beneficial. There is no correct style of research writing. In general, it should attempt to be accurate, impersonal and objective. For example, personal pronouns like ‘We’ (informal language) are used less often than in other writing. So, some sentences need to be revised.

**Response:** Thank you for this suggestion. We have now replaced active voice with passive voice regarding “we” and “our” (informal language) as suggested.

Reviewer #5

Note 1. At Line 72: Authors could write a more clear sentence. (? in 11.2% of shortage case......)

**Response 1:** Thank you for this comment. We have now revised the sentence as follows: “The wholesaler has reported the reason for the shortage to community pharmacies in 11.2% of medicine shortage cases.”

Note 2. Line 78: It is not clear authors were intended to write "Auto-injectors" or "Epinephrine Auto-injectors".

**Response 2:** Thank you for your comment. We have clarified the subtitle and text.

Note 3. Line 122-132: If it reflects the methods of search, should these lines put in 'Methods' Part?

**Response 3:** Thank you for your comment. The literature search was initially done since it justifies our study because of the lack of prior knowledge and literature of epinephrine auto-injectors. The literature search was conducted according to the peer review comments in the first review round. Therefore, it was suggested that the literature search should be put in the section prior to the Methods part.

Note 4. Line 175, 176: If authors intend to justify inclusion of the study period in a sentence "The literature review found......", should it be part of Methods rather than of Purpose?

**Response 4:** Thank you for your comment. We have now clarified the Methods and Purpose part of the text by removing this sentence from Purpose to Methods.

Note 5. In Methods section: It is better to define a. Auto-injectables b. Shortage of auto-injectable. How Shortage of Auto-injectables is defined by Fimea?

**Response 5:** Thank you for your comments. We have checked the correct terms in the literature, and we think that they are “auto-injectors” and “shortage of auto-injectors”. We have provided the definitions for them in the Materials and Methods section. Also, the definition from Finnish Medicines Agency was provided in this section.

Note 6. The readers might not be friendly to interpret points from Table 2. Why many rows for a Auto-injectables like EpiPen is not clear.

**Response 6:** Thank you for this comment. We have now clarified this by adding a footnote under the table. Shortage periods have now been merged in one cell in the table. Also, further information on this table has been added in the text.

Note 7. In Discussion Part: It is expected the following points by authors:
a. Further elaboration of possible reasons for findings on research questions first and second.
b. Association between 'Searching' and 'Shortage': what type of association might be? Can it be temporal or spurious association?

**Response 7:** We thank for these comments.

a. We have elaborated further on possible reasons for findings of research question one and two. First, more information on professionals’ searches has been provided in Discussion. Second, more information on the relation of citizens’ article openings and shortages, as well as summertime and lesser prescriptions, has been added in Discussion.

b. We have now specified the association as “temporal relation” in Discussion and elsewhere in our paper. However, our data on searches and shortages are not able to provide statistical analysis, such as correlation, so we use “visual relation” or “temporal relation” between searches and shortages explored in the figures. We found temporal relation between searches and shortages, thus we mention “visually similar seasonal patterns” in Discussion. Also, this has been added in the Limitations section.
